# Continuous Auditory Feedback of Eye Movements: An Exploratory Study toward Improving Oculomotor Control

**DOI:** 10.3389/fnins.2017.00197

**Published:** 2017-04-25

**Authors:** Eric O. Boyer, Arthur Portron, Frederic Bevilacqua, Jean Lorenceau

**Affiliations:** ^1^STMS Lab, IRCAM - Centre National de la Recherche Scientifique - UPMCParis, France; ^2^Laboratoire des Systèmes Perceptifs, LSP Centre National de la Recherche Scientifique (CNRS), UMR8248, Département d'Etudes Cognitives, Ecole Normale Supérieure-PSLParis, France

**Keywords:** auditory-motor learning, eye movements, smooth pursuit, voluntary oculomotor control, sonification

## Abstract

As eye movements are mostly automatic and overtly generated to attain visual goals, individuals have a poor metacognitive knowledge of their own eye movements. We present an exploratory study on the effects of real-time continuous auditory feedback generated by eye movements. We considered both a tracking task and a production task where smooth pursuit eye movements (SPEM) can be endogenously generated. In particular, we used a visual paradigm which enables to generate and control SPEM in the absence of a moving visual target. We investigated whether real-time auditory feedback of eye movement dynamics might improve learning in both tasks, through a training protocol over 8 days. The results indicate that real-time sonification of eye movements can actually modify the oculomotor behavior, and reinforce intrinsic oculomotor perception. Nevertheless, large inter-individual differences were observed preventing us from reaching a strong conclusion on sensorimotor learning improvements.

## 1. Introduction

Real-time auditory feedback of body movements appears promising in several applications such as sport training and rehabilitation, for instance after a stroke, for children with developmental disorders as in writing (Danna et al., 2013, 2015) or during the course of a neurodegenerative disease (Nicolai et al., 2010; Sigrist et al., 2013). The use of auditory feedback that is generated from real-time movement is often referred to as movement sonification (Bevilacqua et al., 2016). The concept of sonification differs from simple auditory feedback or sound alarms by providing continuous auditory feedback generated concurrently to the action analyzed in real-time. Sonification can inform the users on the movements they execute, who can, in turn, adapt their behavior. This additional information can be used to improve performance, accuracy of executed vs. planned movements, regularity and reliability of gesture, in writing, walking, or grasping for instance. Various sonification strategies have been tested and reported in a growing but still scattered literature. The use of sonification has been experimented with multidimensional data (e.g., whole body movements) and out-of-the-lab scenarios (Roby-Brami et al., 2014). Some strategies aim at informing participants on the success or on the error they make in comparison of a reference movement—which is referred to as knowledge of result (Hartveld and Hegarty, 1996; Rosati et al., 2012). Others sonify movement parameters (e.g., sonifying the velocity of a body part) to help mastering a gesture by providing an additional auditory feedback which characteristics can enhance cognitive access to, and evaluation of, the executed movement—referred to as knowledge of performance (Subramanian et al., 2010; Boyer et al., 2017). Generally, movement sonification is related to movements that participants can overtly control, that is, movements with means to voluntarily modify aspects of a motor plan as a function of the auditory feedback, so as to adjust the subsequently generated movement. Often, the body movements are made under both auditory and visual control.

In this study, we explore the effects of sonifying the movements of the eyes. Eye movements are very peculiar body movements: they occur very frequently, almost never cease, move the visual sensors (the retina), and are thus an intrinsic part of a complex sensory-motor loop. Moreover, the repertoire of eye movements is very rich, ranging from small fixational eye movements, to fast saccades and smooth pursuit eye movements (SPEM). The latter is known to be impossible in the absence of a moving visual, auditory or illusory target to track (Lisberger et al., 1987). Learning strategies aiming at voluntarily mastering eye movements independently from vision therefore require a feedback that can inform individuals in real-time on the fastest, movements that the body can generate. Oculomotor activity being mostly controlled by external visual cues, individuals may decide where and what to look at, or be attracted by some salient features of a visual scene. However, the eye movements needed to attain such a “visual goal” are covertly generated by sub-cortical and cortical structures, that embed a mostly automatic sensory-motor loop, with little conscious choice on how, or at what speed, the eye movements will be performed—but see (Madelain and Krauzlis, 2003). Although proprioceptive and kinesthetic information are used to control the eyes (Gauthier et al., 1990; Ingram et al., 2000), there is little cognitive access to these information which would allow for sensory feedback on the action performed or intended with the eyes. Moreover, there is little feedback available to appreciate whether an eye movement was fully successful or not, as long as the visual target can be discriminated and identified. The visual “reward” might be better appreciated in reading for instance, because access to the meaning of a written word may be impossible if its image is far off the fovea, where visual acuity is best. In case of saccade undershoot or overshoot, however, corrective saccades are quickly produced (Morris, 1984), but we are not aware of studies showing that it is possible to report on the number and/or amplitude of these. Introspection suggests that we know very little of our own eye movements, an intuition confirmed by the results of the present study.

To our knowledge, no study described the usage of real-time and continuous sonification of eye movements for SPEM learning. Sound has been mostly used as a stimulus, especially exploiting the ability of the auditory system to localize sound sources in surrounding space. Gauthier and Hofferer (1976) and then Ward and Morgan (1978) observed that eye pursuit movements can be generated in the dark while following auditory targets moving in front of the participant. More recently, Berryhill et al. (2006) compared different stimuli informing about the motion of a pendulum and measured the tracking gain exhibited by the participants. They compared auditory (loudspeaker attached to the pendulum), tactile (the experimenter moved the pendulum against the subject's arm) and proprioceptive modalities (subjects moved the pendulum themselves). Results showed that tactile and proprioceptive stimuli provide more information for tracking that auditory stimulus, and led to a higher tracking gain. Kerzel et al. (2010) have established a link between auditory perception and catch-up saccades. By observing the decrease of tracking gain and the number of saccades produced after the brief and sudden appearance of distractors while tracking a target, they showed that saccades can be suppressed during a short time after the appearance of a distractor. When the distractor was a loud and task-incongruent sound—a 10 ms white noise click at 83 dB(A)—the tracking gain was also less affected than with a visual distractor appearing at the periphery of the visual field. It is interesting to note that sound has already been used to initiate SPEM, but only as an external stimulus. Madelain and Krauzlis (2003) used pure tones (100 ms *beep*) to notify the production of smooth movements but stopping the sound if the subject produced saccades; this represents a knowledge of performance feedback (KP). A knowledge of result feedback (KR) is also produced (2 *beeps*) in case of success in a trial, in addition to a video animation and money reward. However, in the absence of any interactive aspect of the auditory feedback, motor control is driven one way, only from perception to action without closing the loop.

**Figure 1:**
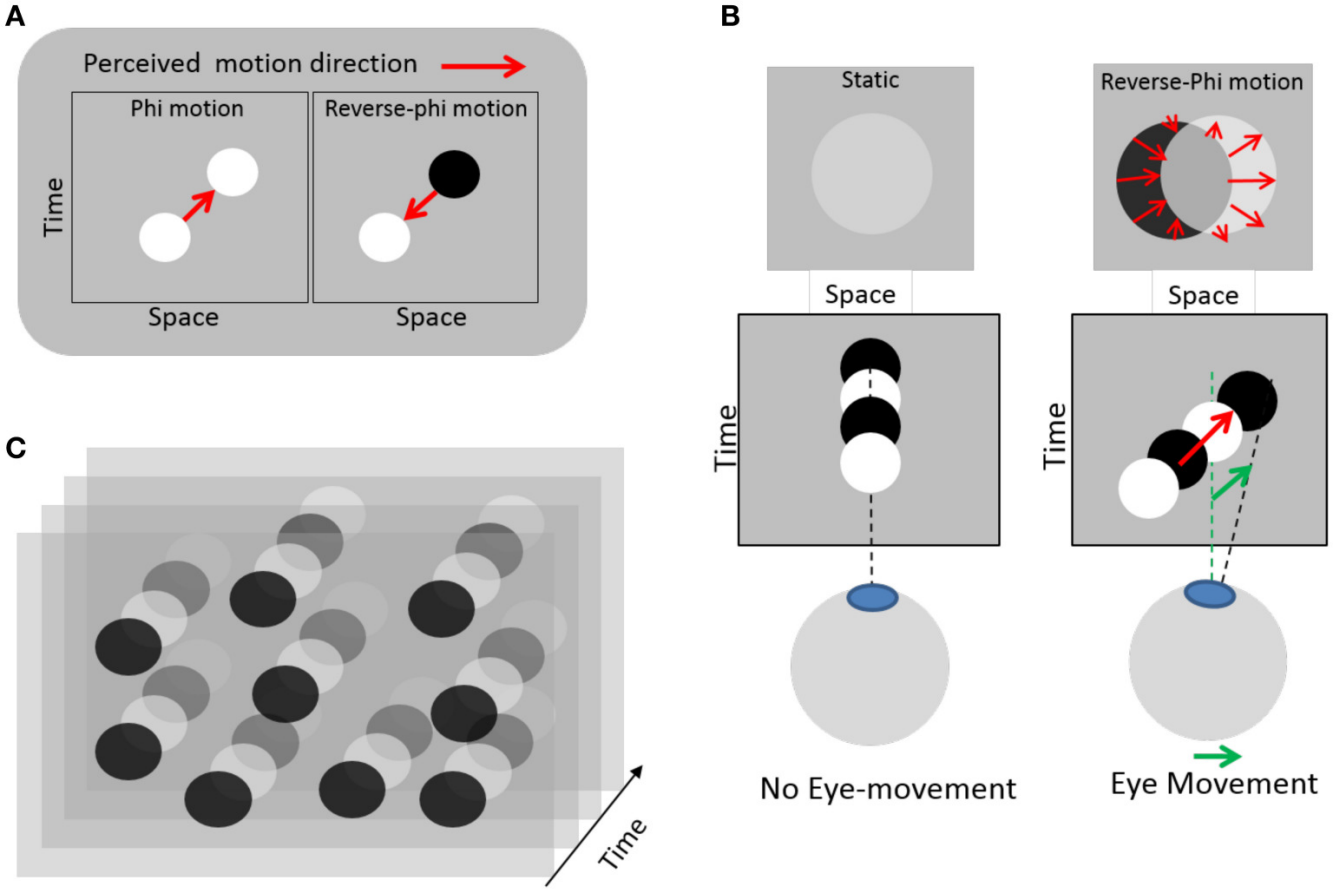
**Illustration of the eye-induced illusory motion used in the experiment. (A)** Left: standard apparent motion elicited by changing a target position between successive frames (red arrow). Right: reverse-phi apparent motion seen in the reversed direction (red arrow) when the target changes contrast polarity between frames (Anstis, 1970). **(B)** Left: with static eyes and a static disk changing contrast polarity, no motion is seen; the disks appear faint or are barely visible. Right: when the eyes move, the disk slips on the retina in a direction opposite to that of the eyes, but this retinal slip elicits an illusory reverse-phi motion in the same direction as the eyes. Relying on this illusory and eye-induced reverse-phi motion provides a visual moving substrate to sustain smooth pursuit eye-movements. **(C)** Static texture of disks changing contrast polarity over time used in the experiment. Reproduced from (Lorenceau, 2012), courtesy of the author.

In this study we use real-time continuous sonification of eye movement (e.g., continuous auditory feedback), which can provide gradual information, within short time scales. One initial hypothesis is that eye movement sonification could enhance proprioceptive feedback during motion and possibly provide a positive reinforcement feedback regarding the production of smooth pursuits. The lack of proprioceptive feedback during eye movement is supposed to be detrimental to free SPEM with reverse-phi, as the necessary visual percept itself is conditioned to movements initiated. For a beginner the lack of proprioceptive feedback could also lead to poor inverse models development for the sensorimotor system. To evaluate the extent to which learning to master one's eye movement would benefit from such sound feedback, we choose to couple eye movements to sound in an experimental protocol comprising two different tasks. Moreover, we attempt to train participants to control SPEM, rather than other eye movements, as this type of movement can hardly be generated in the absence of a moving target. In this way, we ensured that learning, if any, would occur without strong prior cognitive or motor control. To this aim, we employed a visual paradigm where the eye movements themselves entail the perception of visual motion in the same direction as the eye movement (Lorenceau, 2012). This contradicts everyday experience where pursuing a moving target entails a retinal slip of the static background in a opposite direction as the eye movement. It is made possible by relying on a visual illusion known as reverse-phi motion (Anstis, 1970). This illusion occurs when a target (e.g., the frame of a movie) changes its contrast polarity during motion (e.g., alternating positives and negatives frames of a movie). A correlate of this motion illusion was later found in cortical neurons selective to the direction of moving stimulus (middle temporal motion area, MT) that invert their response when stimulated with a target whose contrast polarity alternates over time, as compared to a target moving with constant contrast polarity (Krekelberg and Albright, 2005). In our modified version, static disks randomly distributed periodically change contrast polarity, from darker to brighter relatively to a gray background (Figure 1). Fixating this display without moving the eyes elicits a faint perception of static disks, because the temporal integration of quickly alternating contrasts of opposite polarity entails contrast cancellation. However, whenever an eye movement occurs, the projection of dark and bright disks are spatially offset on the retina, which in turn elicits an illusion of visual motion in the very direction of the eye movement. This perception of motion then feeds the oculomotor pursuit system with a motion to track, on which individuals can rely to voluntarily generate SPEM. With training, individuals can learn to master this visuo-oculomotor loop so as to generate digits, letters or words (Lorenceau, 2012).

## 2. Materials and methods

### 2.1. Participants

Fourteen participants volunteered for the experiment. All declared to be healthy and reported normal hearing. The experiment was conducted in accordance with the Declaration of Helsinki. Written informed consent was obtained from all the participants after complete explanation of the nature of the study which had been approved by the ethics committee of the CPP Ile-de-France VI, Groupe hospitalier Pitié-Salpêtrière. All participants were paid for their time. The experiment lasted for 8 daily sessions of 1 h each. It took place in the LSP lab of Ecole Normale Supérieure of Paris. Six participants had already participated in an experiment involving eye-tracking. One participant had already performed a reverse-phi motion training for about 10 h, 1 year prior to the experiment. Before the main experimental sessions, a preliminary session allowed the identification of participants who were uncomfortable with eye-tracking measurements or who had poor visual tracking ability. To that aim, participants were asked to track a small disk following a predefined path figure over a static background for 35 trials. Afterwards, the experimenter explained the distinction between SPEM and saccadic eye movement to the participants, and introduced the reverse-phi stimulus (see below). Two audio examples of sonified movements were then presented to the participants, one containing mainly smooth pursuit and small catch-up saccades, and one containing many saccades. Participants were asked to indicate which of the 2 recordings was the most successful under the criteria of the experiment, which is to produce as much SPEM as possible. Two participants were withdrawn from the panel following the preliminary session, due to poor pursuit abilities and concentration issues, the final number of participants being 12 (6 female, 6 male, aged 32 ± 15 years old).

### 2.2. Setup and visual stimuli

The experiment took place in a soundproof booth using an Eyelink 1000 eye-tracker (www.sr-research.com). The eye-camera and its infrared light were positioned underneath the stimulation screen, 57 cm away the participants' eyes. The reflection on the retina and the cornea captured by the camera were analyzed by a host computer to provide the absolute gaze position (monocular tracking, left eye). A five-point calibration was performed before each recording, or each time participants moved their head out of the chin rest. The visual stimuli (moving targets and reverse-phi stimulus) were presented on a 1,024 × 768 pixels 60 Hz screen (51,3 × 32,1 cm) facing the participants, and operated by a second computer (HP, Intel Core i7, Windows 7). Data recording includes horizontal and vertical gaze position and pupil diameter (500 Hz sampling frequency). The reverse-phi stimulus consisted in 500 disks (diameter 40 pixels, 2° of visual angle) whose random positioning on the screen was renewed every 50 frames (833 ms), to avoid fixating the shapes they may form. On a single frame, all disks had the same luminance, and all disks changed their contrast polarity at 10 Hz, switching from lighter than the background to darker, and reverse. This flickering stimulus is designed to allow endogenously generating SPEM, as shown in a previous study (Lorenceau, 2012; Portron and Lorenceau, 2017).

### 2.3. Eye movement sonification

A third computer (MacBook Pro, Intel Core 2 Duo, OSX 10.8) received the oculometric data from the EyeLink 1000 at 250 Hz and generated sound feedback in real-time through closed headphones. A custom program has been developed using the eye-tracker built-in API to transmit the eye-tracker data with OpenSoundControl (www.opensoundcontrol.org) and a UDP connection protocol to the third computer. Incoming data were processed with a patch built under the Max/MSP environment (www.cycling74.com). The sonification of the eye data was based on two processes: one for SPEM and one for saccades. The processes generated pursuit and saccade sounds from horizontal and vertical gaze speed signals. Specifically, pursuit sounds were generated from the squared norm of tangential velocity gaze vector. This signal was then filtered by a 20 samples median filter (Bevilacqua et al., 2005). It commanded a resonant filter (Max object *reson* factor *Q* = 10), driven between 100 and 500 Hz, operating on a pink noise. The low end of the spectrum was cut off for clarity. From an ecological point of view, the SPEM sound coupling was designed to evoke the sound of a wind flow, or of rubbing a surface. It was built to be smooth and continuous as well as respecting the dynamic range of the motion. Saccade sounds were generated from the gaze acceleration signal. After computing the squared norm of the acceleration vector, the signal was logarithmically smoothed (20 samples window). The resulting signal commanded a monopole low-pass Filter between 400 and 1,000 Hz filtering pink noise. The envelope of the sound was then shaped with a 5 ms up and a 500 ms linear down-ramp. The average latency of sonification was evaluated around 50 ms. If the velocity of the eye exceeded 100°/s, the saccade sound was triggered, illustrating the saccade profile that was being produced. Below this threshold, no saccade sound was produced. This velocity threshold was set as the upper velocity range of smooth pursuit in humans (Van Donkelaar et al., 1997). Therefore, this saccade sonification was not based on the simple triggering of an audio event. The intensity and the acceleration temporal profile of the saccades were thus preserved and included in the auditory feedback. In this way, this system enabled the participants to perceive the characteristics of the saccades along a continuum, both through loudness and spectral content of the sound. Meanwhile, the pursuit sound was turned off whenever a saccade occurred, using a 50 ms up and 100 ms down linear ramp, in order not to play both the pursuit and saccade sounds simultaneously, since it is impossible to produce both pursuit movement and saccades at the same time.

### 2.4. Training and evaluation protocol

**Figure 2:**
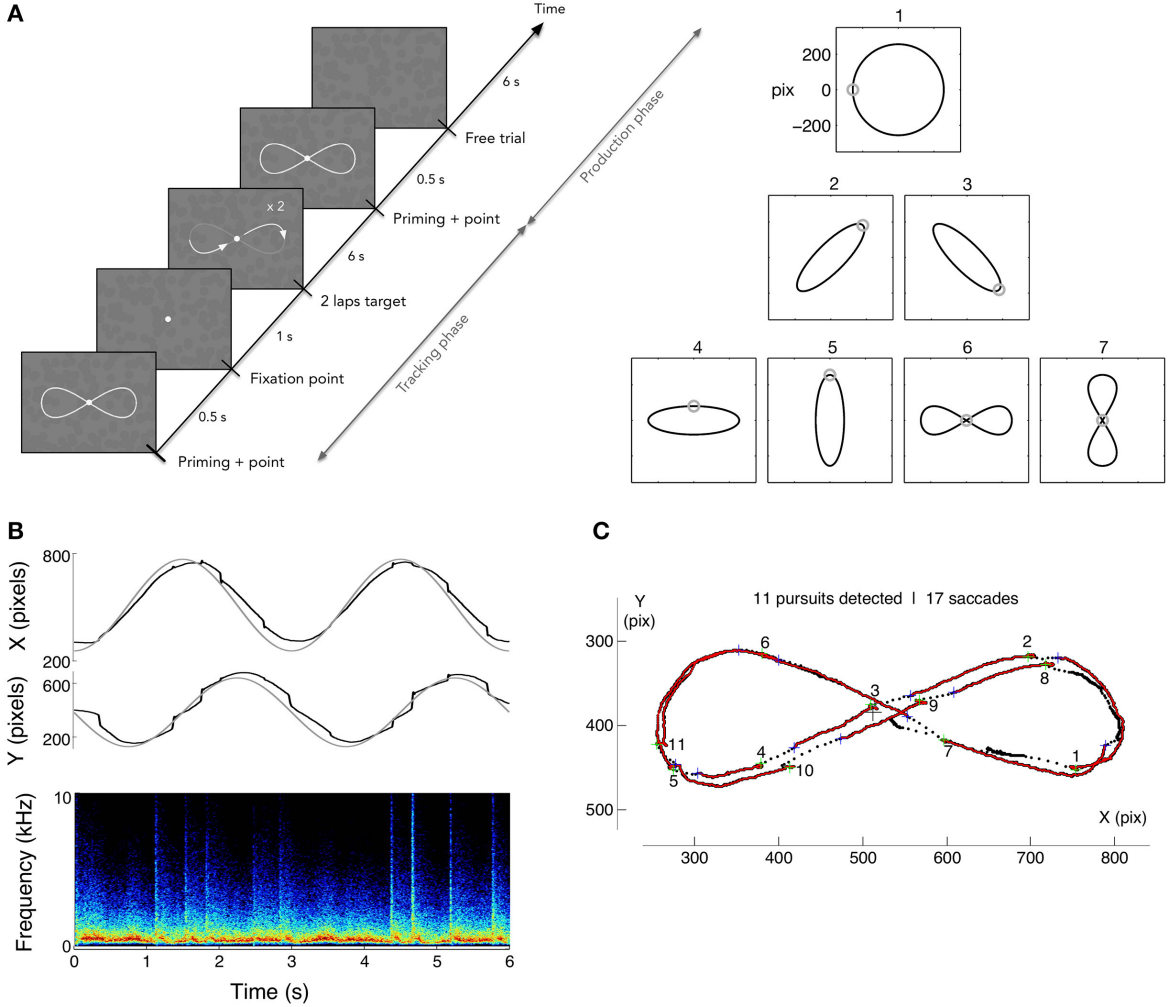
**(A)** Left: time course of a trial in production phase. After showing the randomly chosen pattern for the trial (1 among 7), a dot is shown following 2 loops of the pattern for 3 s each, followed by 2 s with solely the flickering texture. Participants were asked to track the moving target, and to reproduce the previously tracked trajectory on the flickering background, with or without eye-sound feedback. Right: the corpus of pattern trajectories. **(B)** Example of raw eye data (circle pattern) and spectrogram of the subsequent sound feedback where saccades appear as bursts of noise. **(C)** Data analysis consisted in segmenting the pursuits and saccades. In the tracking phase, the velocity gain (ratio of eye velocity to target velocity) and the number of saccades were computed, while pursuit duration and number of saccades were computed in the production phase.

In order to evaluate the potential benefits of sonification on learning to generate smooth pursuit, we designed a dedicated training protocol. The goal was to measure the capability to endogenously produce voluntary smooth pursuits, relying on the illusory reverse-phi motion, and to measure the effects of training, while monitoring how well participants could track a visible moving target. The protocol included a tracking task, follow a moving target describing a variety of motion paths, and the production task, a free pursuit production task smoothly drawing a pattern with the gaze. A set of 7 reference patterns was chosen for both tasks: a circle, four ellipses and two 8-shaped figures. All patterns were inscribed in 512 × 512 pixels area and centered on the screen. The ellipses had an eccentricity of 0.95 and were either vertical, horizontal or tilted at 45°. The 8-shaped figures were closed vertical or horizontal Lissajous curves of parameter *N* = 2. The training protocol was scheduled over 8 daily sessions; four were performed with sonification, and four without. Participants were randomly assigned to one of two groups: group 1 started with sound feedback (Sound First group, denoted SF group thereafter) and finished with four sessions without sound feedback. Group 2 (Sound After, SA group thereafter) did the opposite. Each session was composed of 4 blocks of 5 repetitions of each randomly chosen pattern for a total of 140 trials per sessions. Performing one block took approximately 12 min. A 5 min break was taken between each block. A full data set therefore contained 1,120 trials for each participant. The time course of a trial was as follows (Figure 2): the pattern was first presented for 500 ms together with a dot indicating the starting point of the motion trajectory; the pattern then disappeared and the fixation dot was presented alone for 1 s. After this period, the target dot started describing the pattern for 6 s (2 laps of 3 s each) and then disappeared. The static figure of the pattern was shown again for 500 ms followed by a 6 s period with solely the flickering background. Participants were asked to track the moving target (Tracking phase), and then to reproduce the same pattern (production phase), relying on the flickering background to generate SPEM (see Figure 2A). The mean tangential velocities of the target ranged from 16°/s, for the ellipses, to 21°/s for the circle. The flickering -reverse-phi stimulus- was continuously present throughout a trial. Participants were told they could draw smaller or shifted patterns if they preferred. After each block, participants were asked to evaluate their performance for each pattern, answering the question: “Do you think you smoothly reproduced the pattern?” on a 0-“Not at all” to 10-“Perfectly” scale. At the end of each session, participants filled out a questionnaire to rate their visual and auditory fatigue, from 0-none to 10-maximum.

## 3. Data analysis

### 3.1. Segmentation

The eye position data were low-pass filtered at 100 Hz with a Gaussian filter to reduce noise. A saccade was identified if the instantaneous eye velocity exceeded 100°/s, the same value that was used for the sonification threshold. In the pursuit phase, the gain was computed as the ratio of the eye velocity over the target velocity. Saccades were isolated and the segments between them were counted and analyzed for peak and average velocity, duration and displacement in order to evaluate SPEM. Accumulated eye displacements no greater than 2.5° between two saccades were disregarded. Fixations were detected and taken out if the horizontal and vertical displacements on a segment exhibited a standard deviation smaller than 30% of the 2.5° distance threshold. SPEM consequently matched the three kinematic criteria of speed (<100°/s), effective displacement of the eye (>2.5°) and standard deviation of spatial distribution. Both saccades and pursuit segments smaller than 3 data points (6 ms) were excluded as they were taken for measurement artifacts. An example of segmentation obtained with the method described is shown on Figure 2C for an horizontal Lissajous patter during a tracking task.

### 3.2. Performance evaluation

During the tracking task, performance was assessed by the average gain of tracking pursuits. Specifically, the gain was measured on the pursuit segments validated by the detection algorithm. The number of saccades counted during a trial was also computed. During the production phase, the main evaluation criteria were the cumulative duration of SPEM produced during a trial and the number of saccades. As only few participants were able to reliably control SPEM and reproduce significantly the patterns, it was impossible to quantitatively assess the quality of reproduction of the patterns. Performance during sessions with and without sonification were compared.

## 4. Results

The data set collected in this experiment is large, as the 12 participants underwent 8 sessions, each comprising 4 blocks with 5 repetitions for each of the 7 patterns, with each trial comprising a 6 s tracking phase with the visible moving target (tracking phase), followed by 6 s of pursuit production without the target (production phase). Eye data were analyzed for each trial, separating the two tasks: number of saccades, speed of the saccades, overall duration of pursuits, mean duration of pursuit, longest duration of pursuit, and the pursuit velocity gain for the tracking task. Pupil data were not analyzed. We did not consider the production of micro-saccades and eye tremor, since most data of interest were smooth pursuit and saccade generation. The criteria for identifying saccades and pursuits were the same for all participants (see Section 2).

We focused on the extent to which eye movement sonification helped participants to generate longer and smoother pursuits, as compared to trials without sound feedback. Since participants could improve their performance over time, within a session and across sessions, due to intrinsic learning effects independent of eye sonification, we also considered the evolution of performance, independent of the eye sonification. Below we describe the analysis performed on the different variables derived from the collected data set. Although we observed a slight advantage for the horizontal Lissajous pattern (data not shown), no significant effect of the type of figure was found on SPEM production. Similarly, no significant effect was found on gain or saccade production, therefore we averaged the eye data across all figures for each participant. Similarly, we averaged the data across the 5 repetitions and 4 blocks of each session, in order to obtain tractable mean data between sessions. We report below the results related to sonification and to learning on this averaged data set, separately for the tracking and production phases. We then present the results related to the subjective ratings of performance, before discussing the significance of our findings.

### 4.1. Tracking phase

The typical behavior exhibited by the participants in the tracking phase is a succession of smooth pursuits, of various lengths, interrupted by catch-up saccades. An example of recorded trajectory for pattern 1 (circle) is presented Figure 2. Saccades are visible on the position data, as well as on the spectrogram of the produced sonification under the form of large-band impulses.

**Figure 3:**
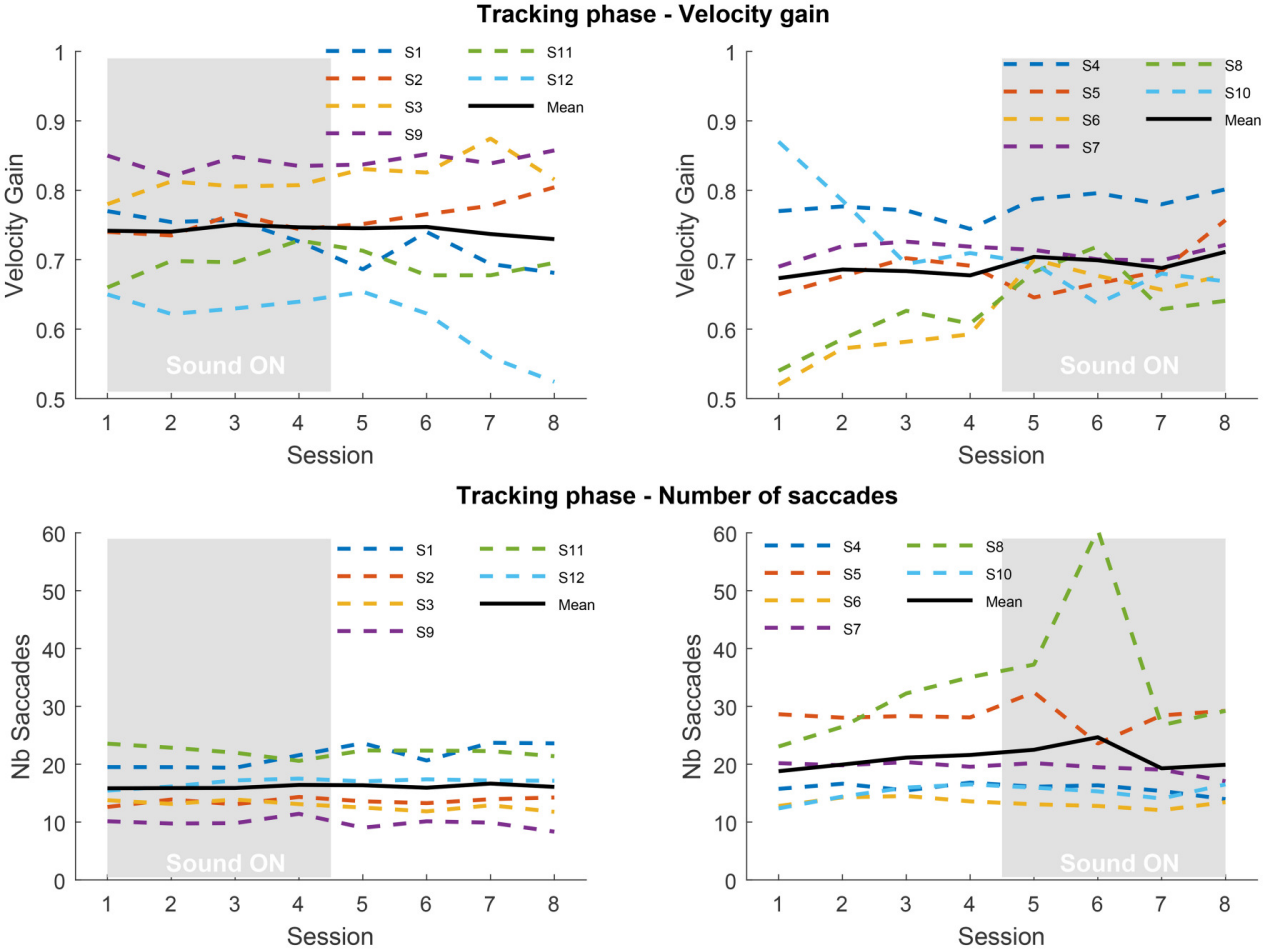
**Top:** velocity gains averaged over repetitions, blocks and pattern trajectories in the tracking phase for the Sound First (SF; **left**) and Sound After (SA; **right**) groups, for each of the 8 sessions. **Bottom:** number of saccades averaged over repetitions, blocks, and pattern trajectories in the tracking phase for the SF **(left)** and SA **(right)** groups. The gray rectangle indicates the sessions performed with sound feedback.

Figure 3 presents the results obtained in the tracking phase, focusing on pursuit velocity gain and number of saccades. For the 12 participants we distinguish those who received sound during the first four sessions from those who received sound in the last four sessions. As it can be observed during the tracking phase the pursuit gain greatly differs across individuals, with some participants being poor trackers (gain below 0.8) and some reaching the gain expected from data found in the literature, above 0.8 (Meyer et al., 1985). Overall, there is little effect of training or sonification on velocity gain, despite the large number of trials, except in some individuals; see for instance the gain increase for participants #6 and #8. Conversely, participant #12 from the SF group, exhibits a decrease of gain when sound is removed. The mean gain during sessions with sound is 0.744 ± 0.073 for SF group and 0.701 ± 0.047 for SA group. During sessions without sound gain is 0.740 ± 0.098 for SF group and 0.680 ± 0.086 for SA group. No statistical analysis could be performed to reliably compare the two groups due to heterogeneity and a too small number of participants. It is difficult to assess the baseline tracking level of each group for the same reasons (also during the inclusion sessions). It appears that both groups could have different baseline tracking abilities, which would make comparisons questionable. The number of intrusive saccades remains stable across the experiment for SF group as well as for SA group, although participants #5 and #8 exhibit more variations. Again, the heterogeneity between participants is substantial with some participants producing three times more saccades than others.

**Figure 4:**
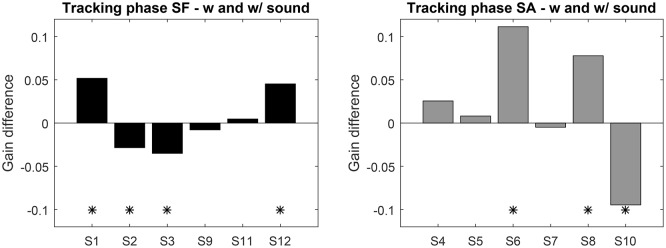
**Top: Average gain difference between the sessions with and without sound feedback (left:** SF group, **right:** SA group). Positive values indicate an increase with sound. “^*^” Indicates significant difference (*t*-test *p* < 0.05).

Instead of comparing groups, we analyzed the performance before and after exposure to sonification for each participant individually. For this, we computed the mean gain difference over the 4 sessions with and without sonification. Sessions gain with and without sound were tested with 95% confidence *t*-test (see Figure 4). Seven participants exhibit a significant evolution of gain with sonification, four of them increased gain with it. The gain differences for SF group never exceed 0.05, whereas the significant differences in SA group reach 0.1. This shows that, if sonification has an effect on pursuit gain, it can affect the gain both positively or negatively, with variable sizes. Also, being exposed to sonification in the first part or the second part of the experiment might play a role as well.

### 4.2. Production phase

**Figure 5:**
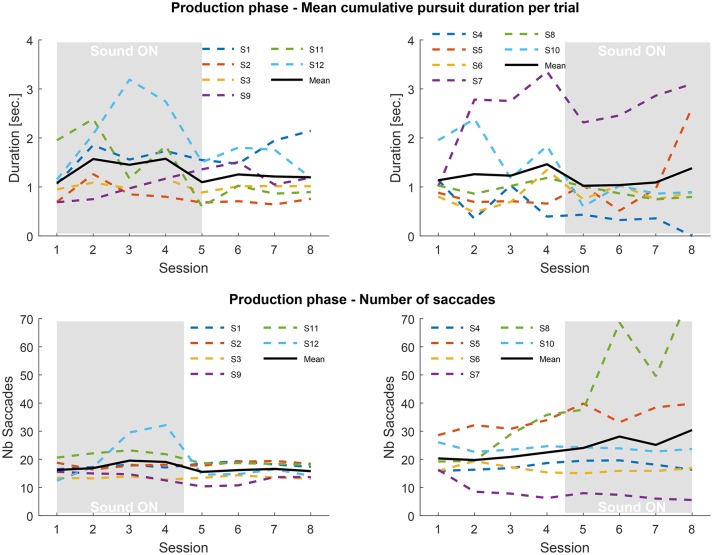
**Top:** mean cumulative pursuit duration per trial averaged over repetitions, blocks and trajectories in the production phase for the SF **(Left)** and SA **(Right)** groups, for each of the 8 sessions. Trials with no pursuits detected are not counted. **Bottom:** number of saccades averaged over repetitions, blocks and trajectories in the production phase for the SF **(Left)** and SA **(Right)** groups. The gray rectangles indicate the sessions performed with sound feedback.

We further analyzed the oculomotor behavior in the production phase, during which participants were trained to endogenously generate SPEM and eye-draw the trajectory of the target previously tracked—see a trial course on Figure 2. As no velocity gain can be computed in this case, because a reference moving target is lacking, we computed the average duration of the pursuits produced, corresponding to the cumulative duration of validated smooth pursuits during the trial divided by the number of pursuits. The trials where no pursuits were produced were taken out of the averages. We also computed the average number of saccades produced in a session (Figure 5).

Once again the inter-individual differences are large, and much more prominent than in the tracking phase. Some observers were able to produce long-lasting SPEM (over 2 s), while others mostly generated saccadic eye movements. On average, the mean duration of smooth pursuit across participants was greater than 1s (SF group: mean: 1.31 s, SD: 0.56 s; SA group: mean 1.32 s, SD: 0.75 s). This finding is in itself a remarkable result, as there is a general agreement to consider that generating SPEM in the absence of a moving target is impossible (Lisberger et al., 1987). As a general rule, whenever a moving target used to initiate pursuit eye movement disappears, the gain of smooth pursuit quickly drops -within 200 ms- after which participants produce saccadic eye movement (Becker and Fuchs, 1985), although the duration can increase with training (Madelain and Krauzlis, 2003). This finding is related to the nature of the flickering background, which can induce an illusion of apparent motion in the direction of the eyes, thus providing a positive visual feedback to the oculomotor system which allows sustaining smooth pursuit for long periods of time (Lorenceau, 2012).

The possible benefit of the sound feedback to maintain smooth pursuit for even longer duration cannot be established for all participants. In the SF group, 2 participants quickly learned to produce very long episodes of pursuit (#1 and #12), while others only showed moderate effects, knowing that learning and sound feedback could have potentially both played a role. This is confirmed by the observation that in the SA group, some participants were also able to generate long episodes of smooth pursuit with no sound feedback.

**Figure 6:**
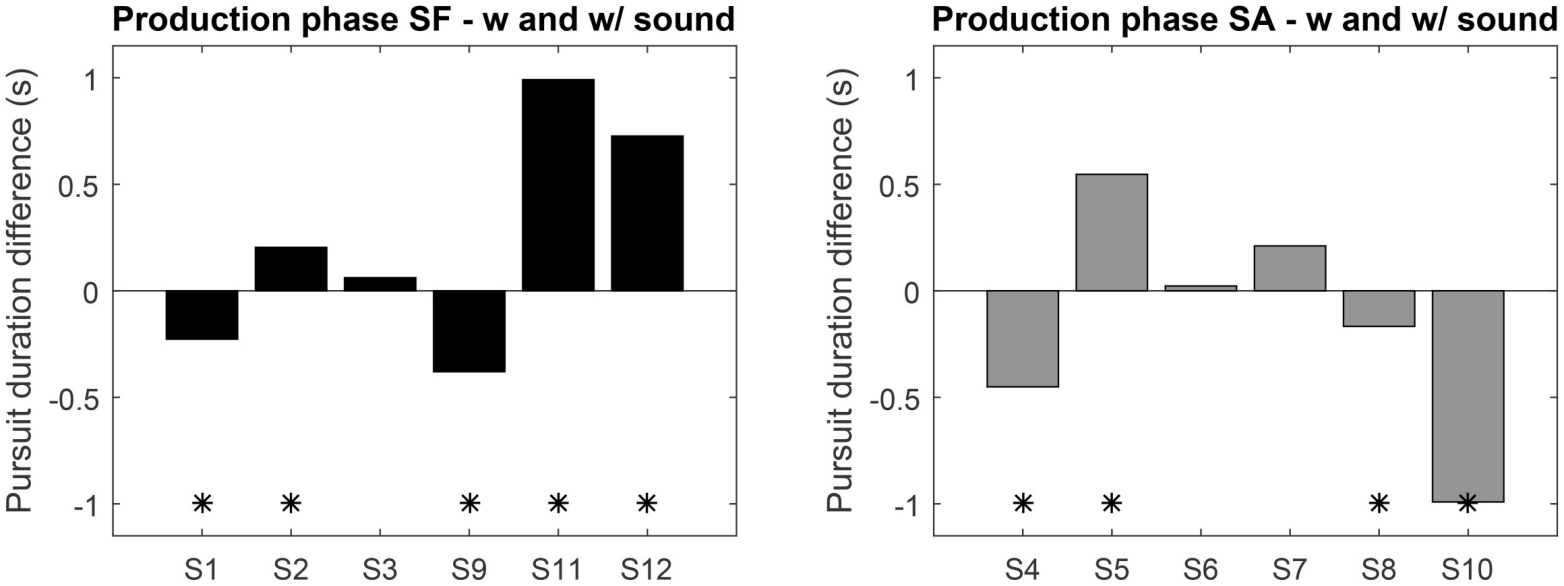
**Average pursuit duration difference between the sessions with and without sound feedback (left:** SF group, **right:** SA group). Positive values indicate an increase with sound. “^*^” Indicates significant difference (*t*-test *p* < 0.05).

During this phase the performance of both groups is closer, only the variance of the SA group is higher. Overall no clear pattern emerges, which points to idiosyncrasies in the mastering of SPEM. Looking at the individual performance (Figure 6) nine subjects exhibited a significant difference in mean pursuit duration between sessions with and without sonification, 4 of them increased the duration with sonification. Averaged difference are about ±1s for both groups. Although large inter-individual differences in mastering eye movements are visible here, these results tend to show that training with sonification could have a measurable impact on individual ocular behavior.

The number of saccades counted during the production phase follows a fairly similar pattern to the tracking phase. The SF group exhibits a steady number of saccades –although participant #12 stands out in sessions three and four, where he also produced many pursuits—throughout the experiment. The SA group shows a slight increase in saccades when sonification comes in (mostly driven by participant #8) with an increasing variance between participants. The presence of sonification in the second half of the experiment for the SA group seems to have an impact on the level of activity of some participants.

In spite of large differences between the participants, the results of the second phase of the experiment also indicate a potential effect of sonification, positive or negative, on the ability to produce SPEM and the level of activity during the tasks (which does not appear correlated).

### 4.3. Subjective ratings and objective smooth pursuit production

**Figure 7:**
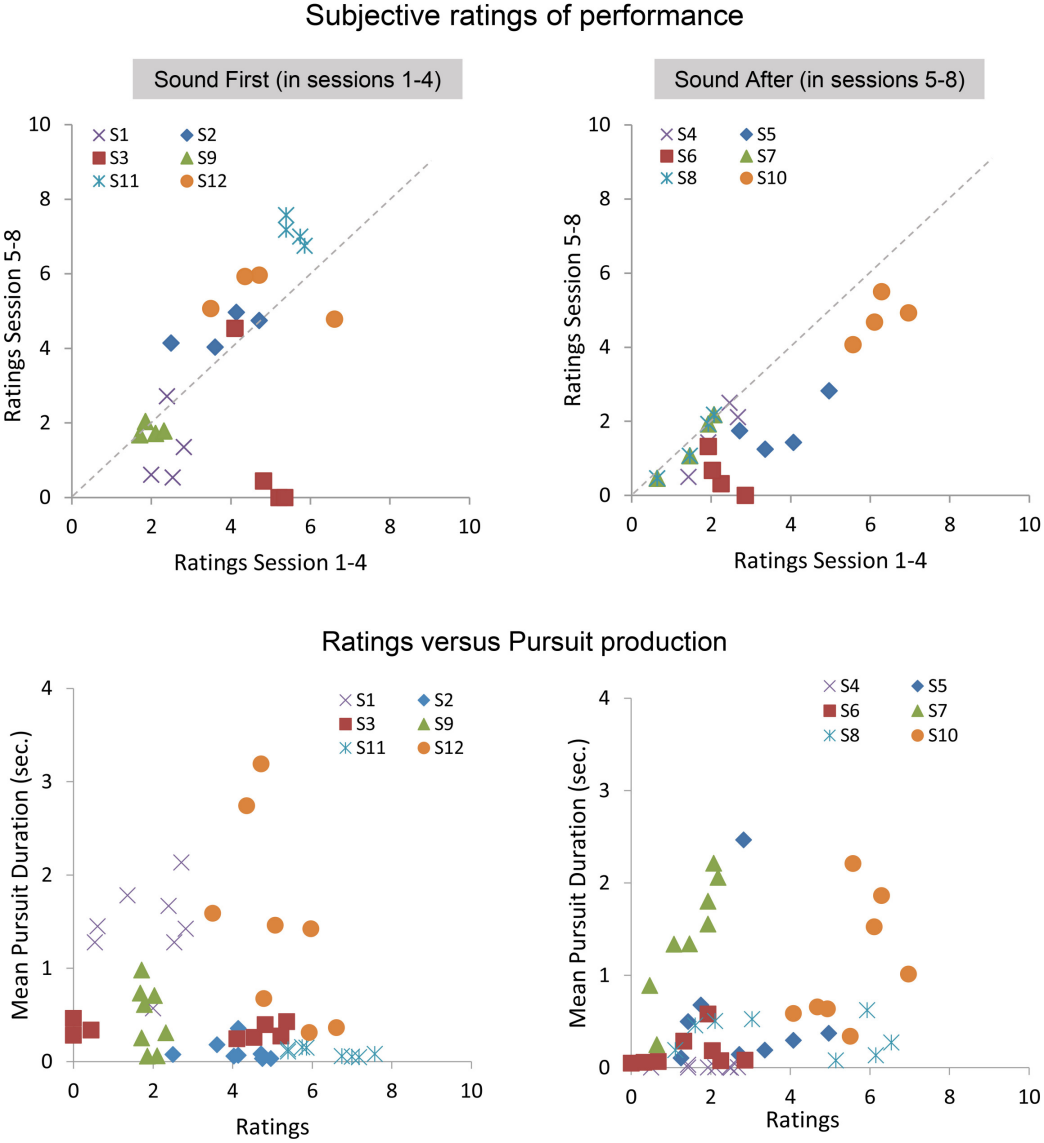
**Subjective ratings averaged across repetitions, blocks, and pattern trajectories in the production phase**. **Top:** average ratings between sessions 5 and 8 as a function of average ratings during sessions 1–4 for the SF **(Left)** and SA **(Right)** groups. **Bottom:** relationships between ratings and the mean pursuit duration produced for the SF **(Left)** and SA **(Right)** groups.

**Figure 8:**
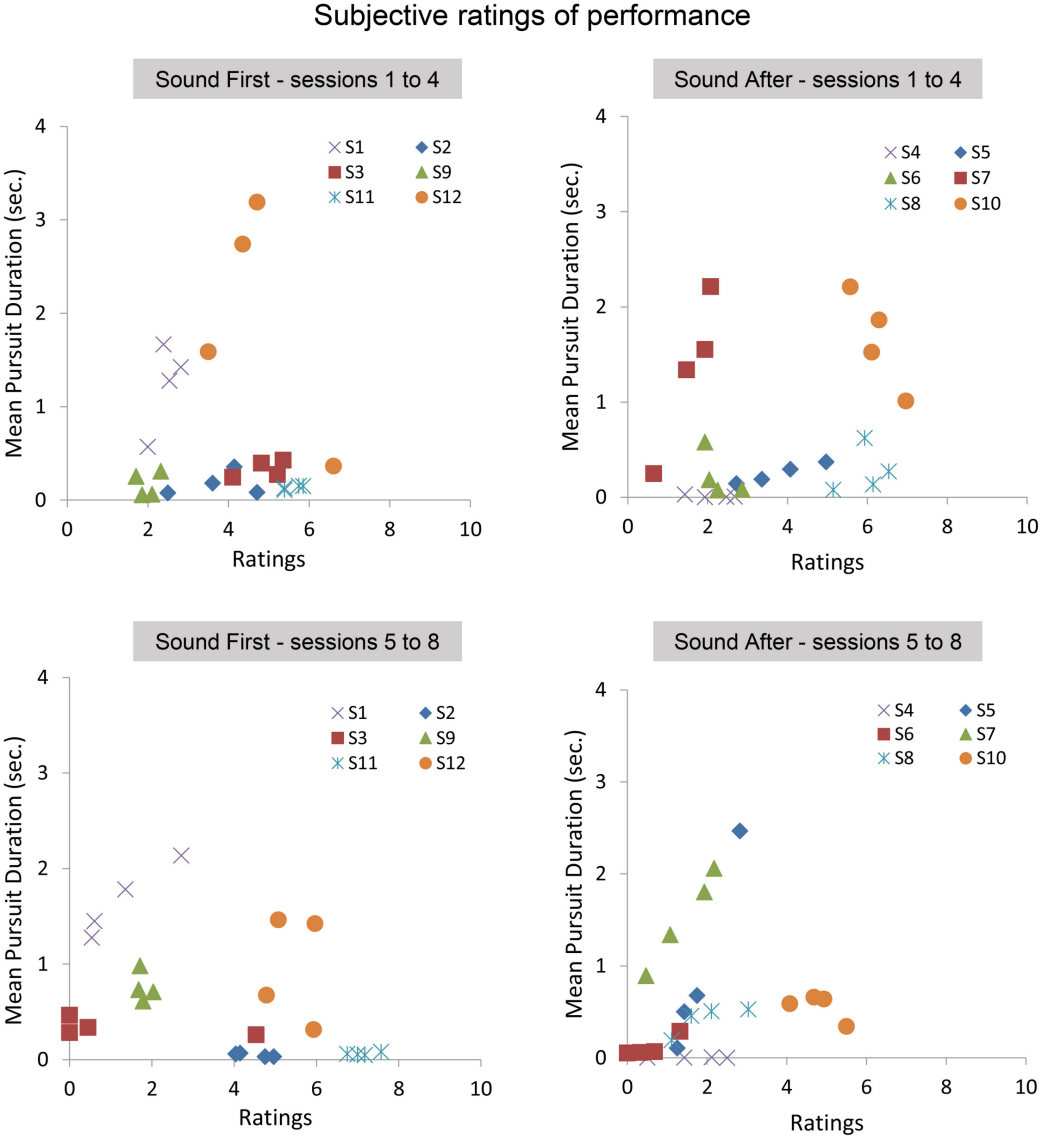
**Subjective ratings averaged across repetitions, blocks, and pattern trajectories. Top:** average pursuit duration as a function of average ratings during the first 4 sessions for the SF **(Left)** and SA **(Right)** groups. **Bottom:** average pursuit duration as a function of average ratings during the last 4 sessions for the SF **(Left)** and SA **(Right)** groups.

At the end of each block, participants gave a subjective assessment of their performance during the block. Below we present the analysis on the average ratings performed with and without sound feedback, and on the correlation between ratings and smooth pursuit production. Figure 7 shows the ratings reported in sessions 1–4 as a function of the ratings given in the sessions 5–8 for the SF and SA group separately. The ratings in the SF group remain stable over sessions, with each participant being consistent in his evaluation (except for participant #3). A noticeable shift is observed in the SA group, with most participants rating their performance as poor or poorer after they performed with sound feedback. Comparing ratings and performance (mean pursuit duration in the production phase, Figure 7 bottom, and Figure 8) provides additional insights into the cognitive evaluation of one's eye movements, and the correlation with mean pursuit duration. In both the SF and SA group, participants' ratings did not reflect their effective performance, except for participant #7, who yet gave low ratings on average.

Overall, the lack of correlation between ratings and pursuit performance indicates that cognitive access to eye movement is poor. However, the difference in ratings between both groups before and after the introduction of the sound feedback suggests that the feedback brought additional information that was taken into account by the participants, especially for the participants of the SA group who discovered their oculomotor behavior through the sound feedback only after the 4 initial sessions.

## 5. Discussion

We present here an exploratory study to use sound coupled to eye movements in order to provide a sensory feedback to an eye activity which is otherwise mostly out of reach of conscious cognition, due to poor proprioceptive and kinesthetic feedback (but see Van Donkelaar et al., 1997; Gauthier et al., 1990). Our hypothesis was that continuous auditory feedback would help participants to master their eye movements in a difficult task involving the endogenous production of SPEM. Despite using a background known to elicit a motion illusion that facilitates sustaining smooth pursuit (Lorenceau, 2012), overall the results did not bring clear-cut evidence that eye-sound coupling helped in this task.

However, there is evidence that eye-sound coupling modifies oculomotor behavior as well as the cognitive evaluation of one's own eye movement performance. We found significant effects of the auditory feedback in the performance of both the tracking and production phase in the majority of participants. Although expected, the effect of such continuous sonification on the oculomotor motor behavior constitutes in a remarkable result, since very few results have yet been reported (Portron et al., 2014). Interestingly, the effects were found to have opposite results depending on the participants. Such variations with participants are often found with movement sonification (Bevilacqua et al., 2016) which create multimodal interactions totally unfamiliar to participant.

In the production phase inter-individuals differences were very large, pointing to both idiosyncrasies in the mastering of eye movements and to, possibly related, differential effects of eye-sound coupling. The sound generated by the eye movements may have been interpreted or listened to differently, depending on the initial level of performance or sensitivity to the sound coupling. Attention to sound might have diverted some participants from the ocular task, or sound might have been perceived as an unavoidable negative feedback. In addition to the expected sensory-motor loop introduced by the sound feedback, a cognitive “interpretative” loop could also have had a detrimental influence, mainly if the sound feedback revealed clearly the poor ability to perform the task; this is visible in the ratings of the participants of the SA group who only discovered how they moved their eyes after session 4. Various participants seem to illustrate these different possibilities, suggesting either different cognitive profiles, and/or different levels of oculomotor control.

Participants who started with sound feedback seemed to exhibit higher gain and production of longer pursuits than the participants who started without it, even during the last sessions performed without feedback. This suggests the existence of a “contextual” effect whereby sonification at the beginning of the sessions provides a knowledge and a meta-cognitive evaluation of one's own eye activity, which can be exploited even after the sound feedback was removed.

We discuss below three points, that might explain the difficulties we found in this study to establish significant positive effects of the sonification on learning the task. First, the number of participants in both groups was limited (*N* = 6). This is a problem given the large inter-individual variability we observe, which prevents from computing reliable between-group comparisons. A second aspect, more technical, must also be considered: the speed of the tracked target was between 16 and 21°/s depending on the patterns, and chosen to allow good tracking in the tracking phase. However, the endogenous generation of smooth pursuit on the flickering background should probably occur at a lower speed to “catch” the illusory motion that allows generating smooth pursuit regarding the stimulus parameters used in this experiment (Lorenceau, 2012; Portron and Lorenceau, 2017). This mismatch between the speed of the tracked target and the eye velocity needed to endogenously generate smooth pursuit can explain, at least in part, the difficulties encountered by participants to freely reproduce the trajectories of the moving target. Moreover, no attempt was made to make the existence of the motion illusion explicit, or to ensure that participants did see -and could exploit- it. We expected that participants would implicitly perceive the motion illusion and use it, but did not evaluated whether this was true.

Finally, the sound used in the present experiment was a filtered pink noise, providing the participant with a noisy sound which average frequency (spectral centroid) varied with the ocular velocity. This choice was motivated to offer a “neutral,” spectrally rich and non-invasive sound, similar to the sound of writing on a hard surface and evoking velocity. Nevertheless, other choices might be more adapted to eye-sound coupling, for instance more clearly pitched or harmonic sound which variation could be easy to discriminate, could offer a better evaluation of one's eye speed. Stereo panning effects or loudness might also be used as additional variables to design the sound coupling (Parseihian et al., 2016). Further in this direction, texture-based sonification (Roby-Brami et al., 2014; Tajadura-Jimènez et al., 2014) offers a wide range of variation of sound attributes which could be coupled to eye movements. Other methods for sonification could also be explored (Bevilacqua et al., 2016).

## 6. Conclusion

Sonification of eye movements is a novel approach to oculomotor control which opens a large field of investigations, both to characterize which parameters are relevant in the large space of possible couplings between eye activity and sound, and to evaluate its interest and usability in a variety of contexts: arts, education, gaming, clinical applications, etc. The results of this first study are encouraging, but also point to the need for large scale studies to better appreciate what characteristics of eye-sound coupling are most efficient, what tasks are the most appropriate to allow improved oculomotor control, and what is the time course of learning needed to use it.

## Author contributions

All authors conceived and designed the experiment together. AP and EB conducted the experiment. AP, EB, and JL analyzed data. EB, JL, and FB wrote most of the manuscript with support from AP. All authors reviewed the manuscript.

### Conflict of interest statement

The authors declare that the research was conducted in the absence of any commercial or financial relationships that could be construed as a potential conflict of interest.
